# The bispecific B7H3xCD3 antibody CC-3 induces T cell immunity against bone and soft tissue sarcomas

**DOI:** 10.3389/fimmu.2024.1391954

**Published:** 2024-05-03

**Authors:** Samuel J. Holzmayer, Kai Liebel, Ilona Hagelstein, Helmut R. Salih, Melanie Märklin

**Affiliations:** ^1^ Clinical Collaboration Unit Translational Immunology, German Cancer Consortium (DKTK); Department of Internal Medicine, University Hospital Tübingen, Tübingen, Germany; ^2^ Cluster of Excellence iFIT (EXC 2180) ‘Image-Guided and Functionally Instructed Tumor Therapies’, Eberhard Karls University Tübingen, Tübingen, Germany

**Keywords:** bispecific antibody, B7-H3, CD276, sarcoma, T cell engager

## Abstract

Sarcomas are rare and heterogeneous malignancies that are difficult to treat. Approximately 50% of patients diagnosed with sarcoma develop metastatic disease with so far very limited treatment options. The transmembrane protein B7-H3 reportedly is expressed in various malignancies, including different sarcoma subtypes. In several cancer entities B7-H3 expression is associated with poor prognosis. In turn, B7-H3 is considered a promising target for immunotherapeutic approaches. We here report on the preclinical characterization of a B7-H3xCD3 bispecific antibody in an IgG-based format, termed CC-3, for treatment of different sarcoma subtypes. We found B7-H3 to be expressed on all sarcoma cells tested and expression on sarcoma patients correlated with decreased progression-free and overall survival. CC-3 was found to elicit robust T cell responses against multiple sarcoma subtypes, resulting in significant activation, release of cytokines and effector molecules. In addition, CC-3 promoted T cell proliferation and differentiation, resulting in the generation of memory T cell subsets. Finally, CC-3 induced potent target cell lysis in a target cell restricted manner. Based on these results, a clinical trial evaluating CC-3 in soft tissue sarcoma is currently in preparation.

## 1 Introduction

Sarcomas account for more than 20% of solid malignancies in children, but less than 1% of solid malignancies in adults (1). They constitute a rare and diverse group of tumors that can arise from bone (BS) or soft tissue (STS) and are classified into more than 100 different subgroups, presenting a unique challenge for diagnosis and treatment (2, 3). The main therapeutic option for BS and STS is surgical removal of the primary tumor, often accompanied by neoadjuvant or adjuvant chemotherapy and/or radiotherapy (4). Radiation therapy particularly plays a role in cases with positive margins after surgery and in high-grade STS to achieve local tumor control (3, 5). Approximately 50% of sarcoma patients develop metastases during the course of disease (6). The 5-year overall survival rate for both bone sarcoma (BS) and soft tissue sarcoma (STS) ranges from 62% to 75%, despite the introduction of new treatment options (7, 8). In particular, the survival rate for patients with high-risk disease or metastasis at the time of diagnosis is only 30%, and new treatment approaches are needed (9).

In recent decades, treatment options for malignant diseases have greatly improved by the introduction of anti-tumor monoclonal antibodies such as rituximab (10). The further improvement of monoclonal antibodies by Fc optimization and the introduction of bispecific antibodies (bsAbs), such as blinatumomab and more recently glofitamab and epcoritamab, is steadily improving survival of patients with hematopoietic malignancies (11–13). When it comes to solid tumors, the introduction of immune checkpoint inhibitors (ICI), such as the anti-PD-1 antibody pembrolizumab, has improved the management of many cancers, particularly advanced melanoma and non-small cell lung cancer, by inducing potent T cell anti-tumor immunity. ICI therapy is currently also being evaluated in several clinical trials for the treatment of STS and is presently approved for certain sarcoma subtypes as part of combination therapies (14, 15). Nevertheless, ICI treatment of sarcomas has a low response rate, highlighting the urgent need for effective immunotherapies (16, 17).

B7-H3 is a transmembrane protein that belongs to the B7 superfamily of immunoregulatory proteins (18). It was shown to affect tumor cell differentiation, invasion, and migration (18, 19). In addition, the B7-H3 complex may enable tumor cells to evade cytotoxic T cell surveillance by acting as a co-inhibitory molecule (20). High expression of B7-H3 was reported to be associated with poor prognosis in patients with different solid tumors such as osteosarcoma or colorectal cancer (21–23). The identification of B7-H3/CD276 expressed by many sarcoma subtypes despite their heterogeneity, fueled interest in this molecule as a target for sarcoma treatment (24–26). In addition, B7-H3 is also expressed on the neovasculature of tumors (25, 27). To target B7-H3 for cancer treatment, we recently developed an optimized bsAb termed CC-3 (28, 29). CC-3 is based on our established IgGsc format, and contains an affinity reduced anti-CD3 binder (30). In preclinical studies, CC-3 demonstrated a favorable safety profile and pronounced efficacy in analyses with multiple gastrointestinal and colorectal cancer cell lines (28, 29). Currently, CC-3 is being evaluated in a phase 1 clinical trial for the treatment of metastatic colorectal cancer (NCT05999396). Here we studied the efficacy of CC-3 for the treatment of multiple sarcoma subtypes.

## 2 Methods

### 2.1 Cell lines

Human cell lines RH30 (rhabdomyosarcoma), SaOs2 (osteosarcoma), SK-LMS-1 (leiomyosarcoma), SW872 (liposarcoma), SW982 (synovial sarcoma) and SW1353 (chondrosarcoma) were obtained from DSMZ or ATCC. All cell lines were cultured in Dulbecco´s Modified Eagle Medium (Thermo Fisher Scientific, Waltham, MA, USA) supplemented with 10% heat-inactivated fetal calf serum (PAN-biotech, Aidenbach, Germany), 100 U/ml penicillin (Merck, Darmstadt, Germany), 100 μg/ml streptomycin (Merck), at 37°C with 5% CO2. All cell lines were routinely tested for mycoplasma contamination.

### 2.2 Isolation of blood cells

Peripheral blood mononuclear cells (PBMCs) from healthy donors were isolated by density gradient centrifugation using Pancoll Cell Separation Solution (PAN-biotech, Aidenbach, Germany). Monocytes were depleted using human CD14 MicroBeads UltraPure Kit (Miltenyi Biotec, Bergisch Gladbach, Germany).

### 2.3 Antibody production

GMP-material of CC-3 used in our clinical trial (NCT05999396) was used for all experiments. The corresponding isotype control (MOPC) was produced as previously described (28, 29). In brief, MOPC was produced in ExpiCHO cells (Gibco, Carlsbad, CA, USA) and subsequently purified from culture supernatant by affinity chromatography on Mabselect affinity columns (GE Healthcare, Munich, Germany). Analytical and preparative size exclusion chromatography was performed using Superdex S200 Increase 10/300GL and HiLoad 16/60 columns (GE Healthcare). Endotoxin levels were measured with EndoZyme II (BioMerieux, Marcy-l’Étoile, France) according to the manufacturer’s instructions and were always < 0.5 EU/ml.

### 2.4 Flow cytometry

Measurements were performed using either a FACS Canto II or FACS Fortessa (BD Biosciences, Heidelberg, Germany), and the data were analyzed using FlowJo-V10 software (BD Biosciences).

#### 2.4.1 B7-H3 expression and antigen shift assay

Sarcoma cells were incubated with CC-3 at the indicated concentrations for 24 h or 72 h. To measure B7-H3 expression, cells were washed and stained with 10 µg/ml anti-B7-H3 antibody (clone 7C4) or corresponding MOPC control, followed by 5 µg/ml donkey anti-human PE conjugate (Jackson ImmunoResearch, West Grove, PA) and analyzed by flow cytometry. The specific fluorescence intensities (SFIs) were calculated by dividing median fluorescence intensity (MFI) obtained with anti-B7-H3by median fluorescence intensity obtained with the isotype control.

#### 2.4.2 T cell activation and degranulation assays

Monocyte-depleted PBMCs were cocultured with sarcoma cells at an effector to target (E:T) ratio of 5:1 with or without CC-3 or MOPC at the indicated concentrations. For analysis of T cell activation, CD69 and CD25 expression were determined after 24 and 72 h, respectively. Cells were stained with CD4-APC (clone L200), CD8a-FITC (clone HIT8a), CD69-PE (clone FN50) and CD25-PE (clone BC96) and the corresponding isotype control in PE (clone MOPC21). To analyze T cell degranulation, cells were cultured for 4 h in the presence of CD107a-PE (clone H4A3). All antibodies were purchased from BD Biosciences (Heidelberg, Germany). Dead cells were excluded by 7-AAD (Biolegend, San Diego, CA, USA).

#### 2.4.3 T cell proliferation and differentiation assays

Monocyte-depleted PBMCs were incubated with sarcoma cells (E:T ratio 10:1) and CC-3 or MOPC (1 nM each). On day 3, cells were restimulated using the same fresh target cell counts and bsAbs as in the initial coculture. On day 6, cells were stained with CD4-FITC (clone OKT4) CD8a-APC-Cy7 (clone SK1), CD45RA-APC (clone HI100), CD45RO-PE-Cy7 (clone UCHL1), CD62L-BV605 (clone DREG 56) and CD197-PE (clone G043H7, all Biolegend, San Diego, CA, USA). Latex beads (Sigma-Aldrich, Darmstadt, Germany) were used to ensure that equal volumes of cell suspension were analyzed. To exclude dead cells from the analysis, fixable violet (Thermo Fisher Scientific, Waltham, MA, USA) was used. To generate t-Distributed Stochastic Neighbor Embedding (t-SNE) plots, equal amounts of CD4^+^ and CD8^+^ T cells for each condition and sarcoma cell line were combined and analyzed for the different treatments.

### 2.5 Cytokine secretion

Monocyte-depleted PBMCs were cultured with sarcoma cells (E:T ratio 5:1) with or without CC-3 or MOPC (1 nM each). After 24 h supernatants were collected and analyzed using the Legendplex Human CD8/NK Panel (BioLegend, San Diego, CA, USA) according to manufacturer´s protocol.

### 2.6 Cytotoxicity assays

Monocyte-depleted PBMCs were cocultured with sarcoma cells (E:T ratio 5:1) in the presence or absence of CC-3 or MOPC (1 nM each) and measured after 72 h by flow cytometry. Tumor cells were labeled with 2.5 µM CellTrace™ Violet (Thermo Fisher Scientific, Waltham, MA, USA). Latex beads were used to ensure equal volumes in each measurement. The xCELLigence RTCA system (Roche Applied Science, Penzberg, Germany) was used to conduct a long-term analysis over 120 hours.

### 2.7 Statistics

Unless otherwise noted, values presented are means ± standard deviation (SD). Continuous variables were analyzed by one-way ANOVA and by Friedman’s test. In cases of normally distributed values where ANOVA revealed significant differences, group comparisons were performed using Tukey’s multiple comparison test. When Friedman’s test showed significant differences for non-normally distributed values, Dunn’s multiple comparison test was used. Statistical analysis was performed with GraphPad Prism (v.9.4.1). All statistical tests were considered significant when the p-value was less than 0.05 (* p<0.05, ** p<0.01, *** p<0.001, **** p<0.0001).

## 3 Results

### 3.1 Clinical relevance of B7-H3 expression in sarcomas

**Figure 1:**
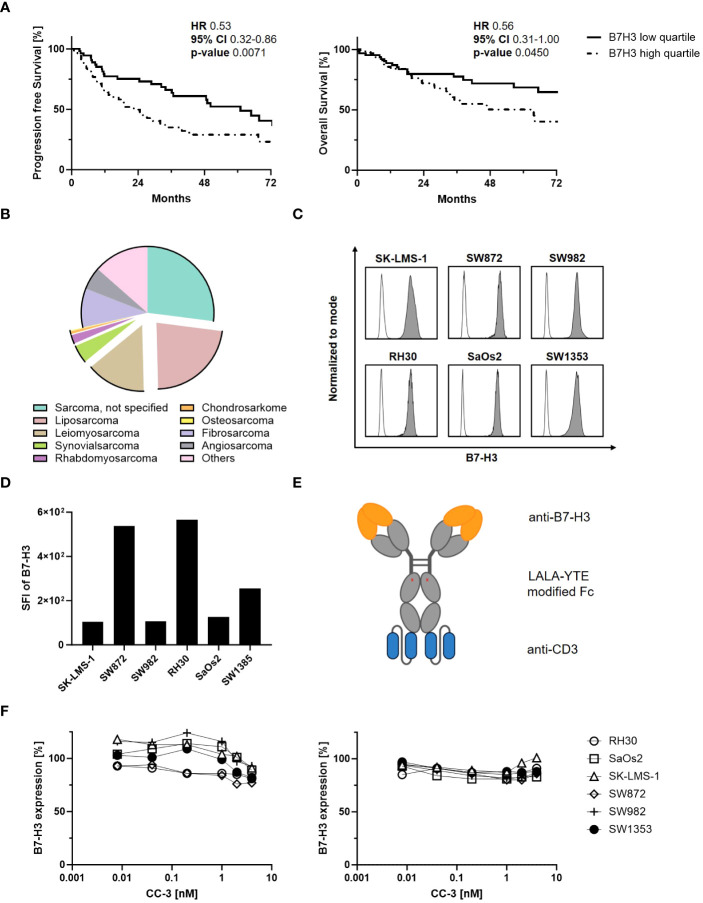
Characterization of B7-H3 expression in sarcomas. **(A)** Sarcoma patients were divided into B7-H3 low and high quartiles according to RNA expression based on data obtained from the The Cancer Genome Atlas (TCGA) program. Clinical data for progression-free survival (PFS, n=231) (left panel) and overall survival (OS, n=259) (right panel) of sarcoma patients were correlated with B7-H3 expression for 72 consecutive months. **(B)** Distribution of sarcoma subtypes diagnosed in Germany in 2013 (n=2438) based on data from Reesing et al, 2018 (31) (CC-BY 4.0 licence). **(C, D)** The indicated sarcoma cells were stained (n=1) with a monoclonal B7-H3 antibody (clone 7C4) followed by an anti-human PE conjugate and analyzed by flow cytometry. **(C)** B7-H3 expression (filled histograms) is shown for the indicated cell lines with corresponding isotype control staining (open histograms). **(D)** SFIs for B7-H3 (n=1), calculated by dividing the MFI of B7-H3 by the MFI obtained with the isotype control, are shown for the different sarcoma cell lines. **(E)** Schematic illustration of the bsAb CC-3 with the anti-B7-H3 (orange) and the anti-CD3 (blue) targeting regions. **(F)** Sarcoma cells were incubated with the indicated concentration of CC-3 for 24 h (left panel) or 72 h (right panel). Cells were then washed and directly reincubated with anti-B7-H3 (clone 7C4) followed by an anti-human PE antibody and measured by flow cytometry. Relative surface expression of B7-H3 was calculated by defining the MFI of cells preincubated without CC-3 as 100% (n=1, performed in duplicate). HR: hazard ratio, 95% CI: 95% confidence interval.

It has been reported that B7-H3 overexpression is associated with cancer progression and poor patient survival (26). We here correlated B7-H3 expression levels with clinical outcomes in sarcoma patients (n=259) using the TCGA database. We observed a significant difference in median progression-free survival between the B7-H3 high quartile (22 months) and the B7-H3 low quartile (61 months) (**Figure 1A**). In addition, the median overall survival for the B7-H3 high quartile was 64 months, while the B7-H3 low quartile did not reach the median survival within the six-year period of data collection. The histological distribution of relevant sarcoma subtypes diagnosed in Germany in 2013 (n=2438), as reported by Reesing et al. in 2018, is simplified in **Figure 1B** (31). While the majority of sarcomas were not histologically defined (27%), described subtypes were liposarcoma (23%), leiomyosarcoma (15%), fibrosarcoma (9%), angiosarcoma (6%), synovial sarcoma (4%), rhabdomyosarcoma (2%), chondrosarcoma (0.6%), osteosarcoma (0.2%) and other subtypes (14%). In our study, we selected sarcoma cell lines representing a total of 44% of the sarcomas analyzed. Binding of CC-3 to tumor cells was observed in all sarcoma cell lines tested, with RH30 showing the highest and SK-LMS-1 the lowest expression levels (**Figures 1C, D, E**). By binding to the target antigen, antibodies can alter its expression on the target cells, which may impact therapeutic efficacy (32). To study whether and how CC-3 binding affected B7-H3 expression on sarcoma cells, all cell lines were incubated with increasing concentrations of CC-3 for 24 and 72 h and then compared to unexposed controls with regards to CC 3 binding. After 24 hours, CC-3 treatment reduced B7-H3 expression by 10% in SK-LMS-1 and by 23% in SW872, which showed the highest reduction at concentrations above 1 nM, (**Figure 1F**, left panel). After 72 h, B7-H3 levels decreased by an average of 11%, with a maximum decrease of 17% in SaOs2, while no changes were observed in SK-LMS-1 (**Figure 1F**, right panel). Additionally, no turnover of target cells without effector cells was observed (**Supplementary Figure 1**).

### 3.2 T cell activation with CC-3

**Figure 2:**
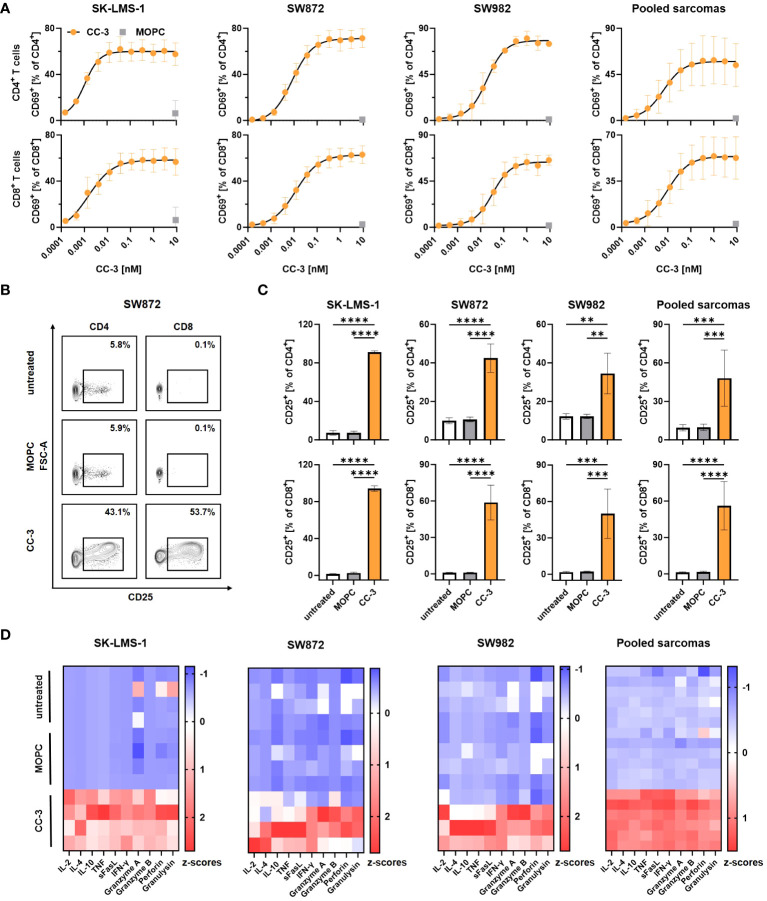
Induction of T cell activation and cytokine release with sarcoma cell lines by CC-3. PBMCs (n=4) were incubated with the indicated sarcoma cells (E:T 5:1) in the presence or absence of CC-3 or MOPC. Unless otherwise noted, all constructs were used at 1 nM. T cell activation by CD69 and CD25 was assessed for CD4^+^ and CD8^+^ T cells by flow cytometry after 24 h and 72 h, respectively, and secretion of cytokines and effector molecules was determined using Legendplex assays after 24 h. **(A)** Activation of CD4^+^ (top panels) and CD8^+^ T cells (bottom panels) was determined by CD69 expression. Combined data from all tested cell lines are shown (right panel). **(B, C)** Activation of CD4^+^ and CD8^+^ T cells was determined by CD25 expression. **(B)** Exemplary results obtained with SW872 cells are shown. **(C)** CD4^+^ (top panel) and CD8^+^ (bottom panel) T cells were analyzed for CD25. Combined data of all cell lines tested are shown (right panels). **(D)** Cytokine and effector molecule release for the indicated cell lines with or without CC-3 or MOPC control after 24 h was determined using Legendplex assays. Combined data from all tested cell lines are shown (right panel). The values presented are means ± SD (* p<0.05, ** p<0.01, *** p<0.001, **** p<0.0001).

To determine the CC-3 dose required to fully activate T cells in cocultures with sarcoma cells, we titrated CC-3 and determined expression of the activation marker CD69 on T cells by flow cytometry after 24 h. CC-3 induced a dose- and target-cell dependent activation of both CD4^+^ and CD8^+^ T cells, whereas the MOPC control showed no effect, even at the highest concentration of 9 nM (**Figure 2A**, **Supplementary Figures 2A, B**). Maximum T cell activation was observed at 1 nM, which was selected as dose for subsequent experiments. Effective cancer treatment, particularly in patients with a high tumor burden, requires a persistent and long-lasting T cell response. To confirm ongoing T cell activation, we evaluated expression of the mid-term activation marker CD25 on T cells after CC-3 treatment by flow cytometry after 72 h of coculture with sarcoma cells. CC-3 treatment resulted in significant and sustained activation of CD4^+^ and CD8^+^ T cells with all sarcoma subtypes tested. The MOPC control showed no difference compared to PBMCs without antibody treatment (**Figures 2B, C**, **Supplementary Figure 2C**). Activation of T cells results in the release of activating cytokines and effector molecules into the culture supernatant. This was analyzed by Legendplex assays after 24 h. Despite a substantial donor variability, treatment with CC-3 resulted in significant increases in IL-2, IL-4, IL-10, and IFNy as well as enhanced release of sFasL, TNF, granzyme A, granzyme B, perforin and granulysin compared to MOPC control in all experiments (**Figure 2D**, **Supplementary Figure 2D**).

### 3.3 Induction of T cell proliferation and differentiation

**Figure 3:**
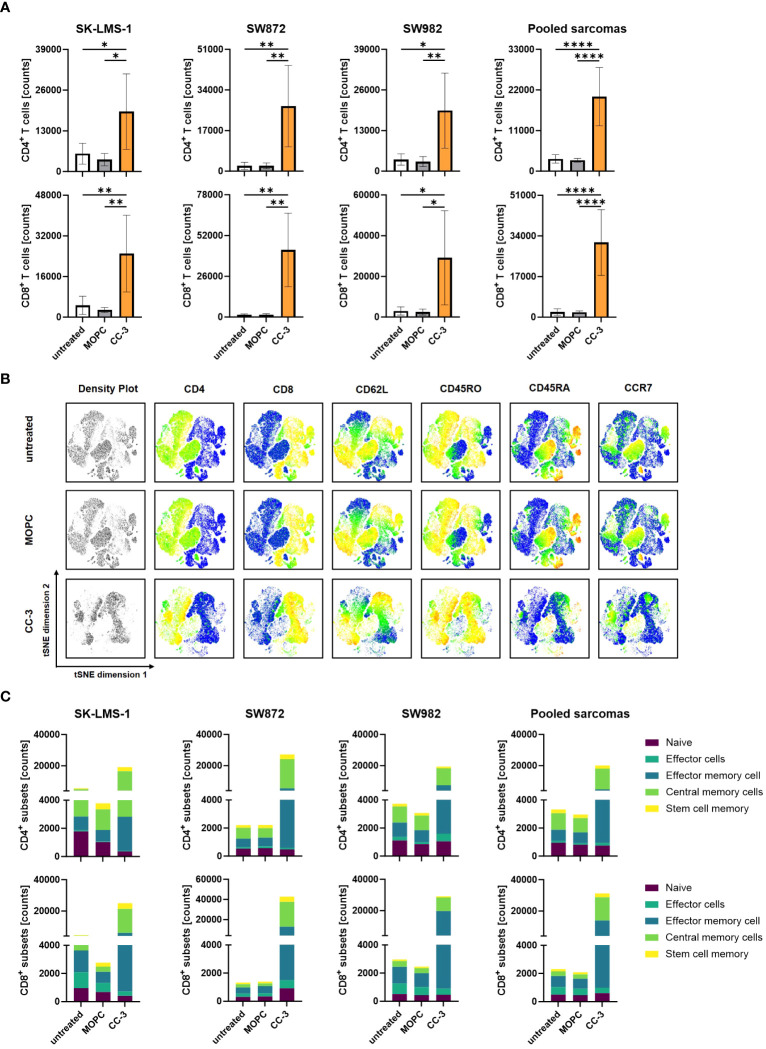
Proliferation and differentiation of T cell subsets after treatment with CC-3. PBMCs (n=4) were incubated with the indicated sarcoma cells (E:T 10:1) in the presence or absence of CC-3 or MOPC control (1 nM each) for 6 days. On day 3, PBMC were re-exposed to fresh target cells and the respective treatment for additional 3 days. On day 6, proliferation and T cell subsets were analyzed by flow cytometry for CD62L, CD45RO, CD45RA and CCR7. **(A)** CD4^+^ and CD8^+^ T cell counts for the indicated cell lines are shown. **(B)** Representative t-distributed stochastic neighbor embedding (tSNE) plots are shown with equal amounts of CD4^+^ and CD8^+^ T cells from each PBMC donor after coculture with all sarcoma cell lines tested. The tSNE visualizes the density of all analyzed T cells, and the expression of each individual marker is displayed in pseudocolor. **(C)** Quantification of CD4^+^ and CD8^+^ T cell subpopulations after coculture with the indicated cell lines or the combined data for all cell lines tested. The values presented are means ± SD (* p<0.05, ** p<0.01, *** p<0.001, **** p<0.0001).

To effectively combat high tumor burden, it is essential to induce T cell proliferation, resulting in a greater number of effector cells available to fight the tumor. We investigated whether and to what extent CC-3 induced T cell proliferation by coculturing PBMCs with and without CC-3 or MOPC control in cocultures with sarcoma cells. On day 3, fresh sarcoma cells as well as CC-3 or MOPC control were added. On day 6, the number of CD4^+^ and CD8^+^ T cells was determined by flow cytometry (**Figure 3A**, **Supplementary Figure 3A**). On average, treatment with CC-3 resulted in a 6-fold increase in CD4^+^ T cell counts and a 15-fold increase in CD8^+^ T cell counts. In contrast, the MOPC control did not alter the number of CD4^+^ or CD8^+^ T cells, confirming that CC-3 mediated its effect on T cells in a target cell-restricted manner. Memory T cells play a critical role for a strong and long-lasting T cell response, as they are able to proliferate rapidly upon reactivation. We investigated the differentiation of T cells induced by CC-3 into subsets of naive cells (CD45RA^+^CD45RO^-^CCR7^+^CD62L^+^), stem cell memory (SCM) (CD45RA^+^CD45RO^+^CCR7^+^CD62L^+^) central memory cells (CMC) (CD45RA^-^CD45RO^+^CCR7^+^CD62L^+^), effector memory cells (EMC) (CD45RA^-^CD45RO^+^CCR7^+^CD62L^-^) and effector cells (CD45RA^+^CD45RO^-^CCR7^-^CD62L^-^) (33). The tSNE visualization technique was used to display the multidimensional flow cytometry data (**Figure 3B**). The population composition obtained with the MOPC control was found to be similar to that of untreated T cells in coculture with sarcoma cells. In contrast, CC-3 specifically stimulated the formation of CMC and EMC in CD4^+^ and CD8^+^ T cells (**Figure 3C**, **Supplementary Figures 3B, C**). Thus, CC-3 shifts the proportions towards CMC in CD4^+^ T cells and higher proportions of both CMC and EMC in CD8^+^ T cells (**Supplementary Figure 3C**).

### 3.4 CC-3 potently induces sarcoma cell lysis by T cells

**Figure 4:**
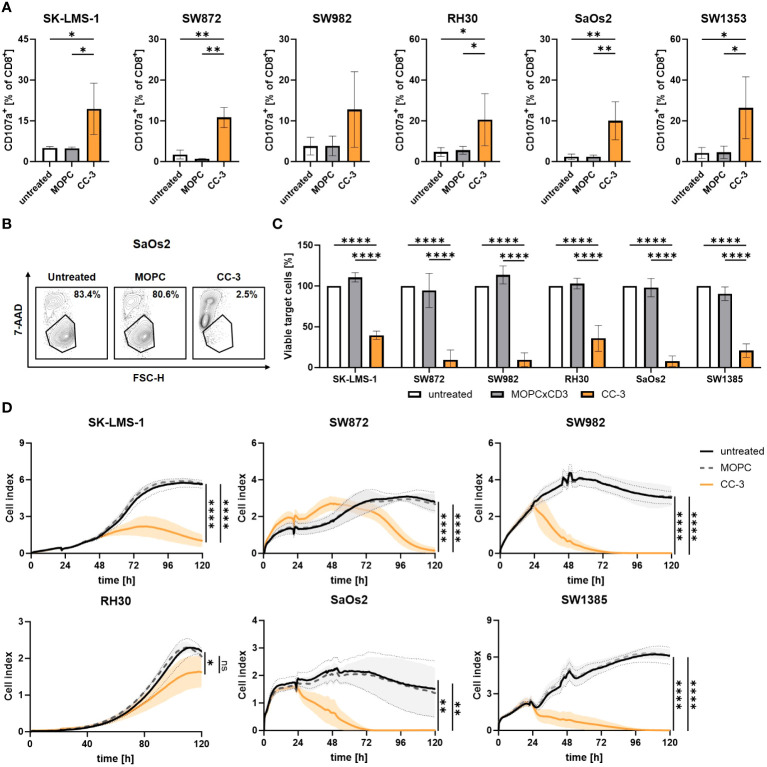
Cytotoxic T cells show effector function after CC-3 stimulation. PBMCs (n=4) were incubated with the indicated sarcoma cell lines (E:T 5:1) in the presence or absence of CC-3 or MOPC control (1 nM). **(A)** CD8^+^ T cells were analyzed for degranulation by CD107a after 4 h of coculture. **(B, C)** Sarcoma cell lysis after 72 h was determined by flow cytometry. Tumor cell counts of untreated cells were set to 100%. **(D)** Long-term cytotoxic effects of PBMCs (n=4) against the indicated sarcoma cell lines were measured by xCELLigence for 120 h. The values presented are means ± SD (* p<0.05, ** p<0.01, *** p<0.001, **** p<0.0001).

When bsAbs bind to tumor cells and stimulate T cells via CD3, cytotoxic T cells release effector molecules such as perforin and granzymes from their secretory granules as a major killing mechanism (34). Degranulation of T cells was analyzed by CD107a expression after 4 h of coculture of - PBMCs and sarcoma cells, with and without CC-3 or MOPC control, by flow cytometry. We found that CC-3 potently stimulated the degranulation of CD8^+^ T cells in cocultures with all sarcoma cells used (**Figure 4A**). Since efficient cell lysis is essential for effective tumor control, sarcoma cell lysis in coculture with PBMCs was determined by flow cytometry after 72 h, with and without CC-3 or MOPC control. CC-3 induced pronounced tumor cell lysis with all sarcoma subtypes, whereas the MOPC control had no effect compared to untreated samples (**Figures 4B, C**). Continuous analysis of the cytotoxic effect over a period of 120 h using xCELLigence confirmed a sustained long-term cytotoxic effect of CC-3 with all sarcoma cells, resulting in significantly reduced tumor burden (**Figure 4D**).

## 4 Discussion

For many years, effective treatment options for sarcomas were lacking, and few improvements were introduced in routine care. Clinical trials including sarcoma patients are complicated by the substantial heterogeneity and rarity of the disease. This also holds true for the various immunotherapies that have revolutionized treatment in other cancer entities in recent years. For example, pembrolizumab and nivolumab are currently being investigated for use in sarcoma patients, but efficacy appears rather limited, in particular when compared to e.g. melanoma or lung cancer (14, 35). One reason may be the very low mutation rate in sarcomas, resulting in low level expression of tumor-specific neoantigens (36). Therefore, there is a high need to develop new therapies for sarcomas that stimulate targeted anti-tumor immunity.

B7-H3 has been shown to influence tumor cell differentiation, invasion, migration, and is discussed to play a role in cancer stemness (19, 37). B7-H3 overexpression was found to associate with tumor progression and aggressiveness in clear cell renal cell carcinoma and osteosarcoma (38, 39). In line, our findings obtained upon analysis of the TCGA database showed that sarcoma patients with high B7-H3 expression experience shorter progression-free and overall survival. B7-H3 was found to be highly expressed in several bone and soft tissue sarcomas, making it a promising target for therapy (26). Sarcomas are generally considered to be quite resistant to immunotherapy, in part due to low immune cell infiltration (16, 17, 40). B7-H3 is not only expressed on cancer cells themselves, but also on tumor vessels. Targeting the latter can be expected to generate a local inflammatory milieu that enhances immune infiltration into the tumor. In addition, damage to tumor vasculature per se would inhibit tumor growth (41). Our study indicates that even after prolonged exposure to high levels of anti-B7-H3 antibodies, a stable antigen expression is prevalent, and this was observed across different sarcoma subtypes. Our data further clearly show that targeting B7-H3 with our bsAb CC-3 induces potent and target-restricted activation of CD4^+^ and CD8^+^ T cells that results in pronounced tumor cell killing, but also T cell proliferation that is particularly important to combat high tumor burden. In addition, CC-3 induced the development of memory T cell subsets such as EMC and CMC, which are essential for effective tumor control (42, 43).

In previous studies, we and others have already shown that monospecific B7-H3 mAbs which recruit NK cells as effector cells are effective in targeting solid tumor cells such as sarcoma, breast and ovarian cancers *in vitro* (26, 44, 45). BsAbs such as CC-3 may mediate enhanced efficacy by recruiting T cells, which exhibit profoundly higher effector potential than NK cells. Additionally, CC-3 contains an optimized antigen epitope with respect to membrane proximity improving tumor lysis and a low-affinity CD3 binder to reduce side effects (28). Several bsAbs targeting B7-H3 are currently under clinical evaluation, such as XmAb808, a B7H3xCD28 bsAb that has been evaluated in advanced solid tumors (NCT05585034). MGD009, a B7H3xCD3 bsAb in a dual affinity re-targeting (DART) format, has been evaluated in various solid tumors including sarcomas. Notably, the clinical evaluation of the MGD009 bsAb was temporarily paused due to a transient elevation of liver enzymes in treated patients. However, the study was resumed following a safety evaluation. It is important to note that bsAbs carrying many single chains tend to aggregate, resulting in off-target T cell activation and cytokine release, but further comprehensive information on MGD009 is not currently available and therefore a direct comparison with CC-3 is not possible.

Our bsAb CC-3 generated in the IgGsc format, which is currently tested in a clinical trial in colorectal cancer patients (NCT05999396), has shown reduced side effects and toxicity in preclinical studies (28–30). In addition to bsAbs, chimeric antigen receptor (CAR) T cells targeting B7-H3 in sarcomas are currently in development (NCT04483778, NCT04897321) (46, 47). However, CAR-T cells require a complex and individualized manufacturing process for each patient, increasing the cost of therapy tremendously. Notably, the actual CAR-T therapy starts a few weeks after the diagnosis, and valuable time is spent on less effective bridging therapies. In contrast, antibody therapy is readily available off the shelf.

In summary, our bsAb CC-3 shows promise as an immunotherapeutic agent for multiple sarcoma subtypes and will be evaluated in an upcoming clinical trial in patients with soft tissue sarcoma. If successful, CC-3 would provide a new and effective treatment option for patients who so far do not have access to advanced and effective immunotherapeutic treatment options for cancer.

## Data availability statement

The raw data supporting the conclusions of this article will be made available by the authors, without undue reservation.

## Ethics statement

The studies involving humans were approved by ethics committee at the Medical Faculty of the Eberhard Karls University and the University Hospital Tübingen (reference number 13/2007V). The studies were conducted in accordance with the local legislation and institutional requirements. The participants provided their written informed consent to participate in this study.

## Author contributions

SH: Conceptualization, Data curation, Formal analysis, Investigation, Visualization, Writing – original draft, Writing – review & editing. KL: Data curation, Formal analysis, Investigation, Visualization, Writing – original draft. IH: Methodology, Resources, Writing – review & editing. HS: Conceptualization, Funding acquisition, Resources, Supervision, Writing – review & editing. MM: Conceptualization, Funding acquisition, Project administration, Supervision, Writing – original draft, Writing – review & editing.
