# Supplementary material for: The bispecific B7H3xCD3 antibody CC-3 induces T cell immunity against bone and soft tissue sarcomas
Source: Front Immunol. 2024 May 3;15:1391954. doi: 10.3389/fimmu.2024.1391954 (PMC11099233; doi:10.3389/fimmu.2024.1391954)
Supplement: Supplementary file 1 [file DataSheet_1.docx]

Supplementary Figures

**Supplementary Figure 1 CC-3 in culture with sarcoma cells.** Sarcoma cell lines were cultured with or without CC-3 (4 nM) (n=1 in duplicates). The number of viable cells was determined by flow cytometry after 72 hours.


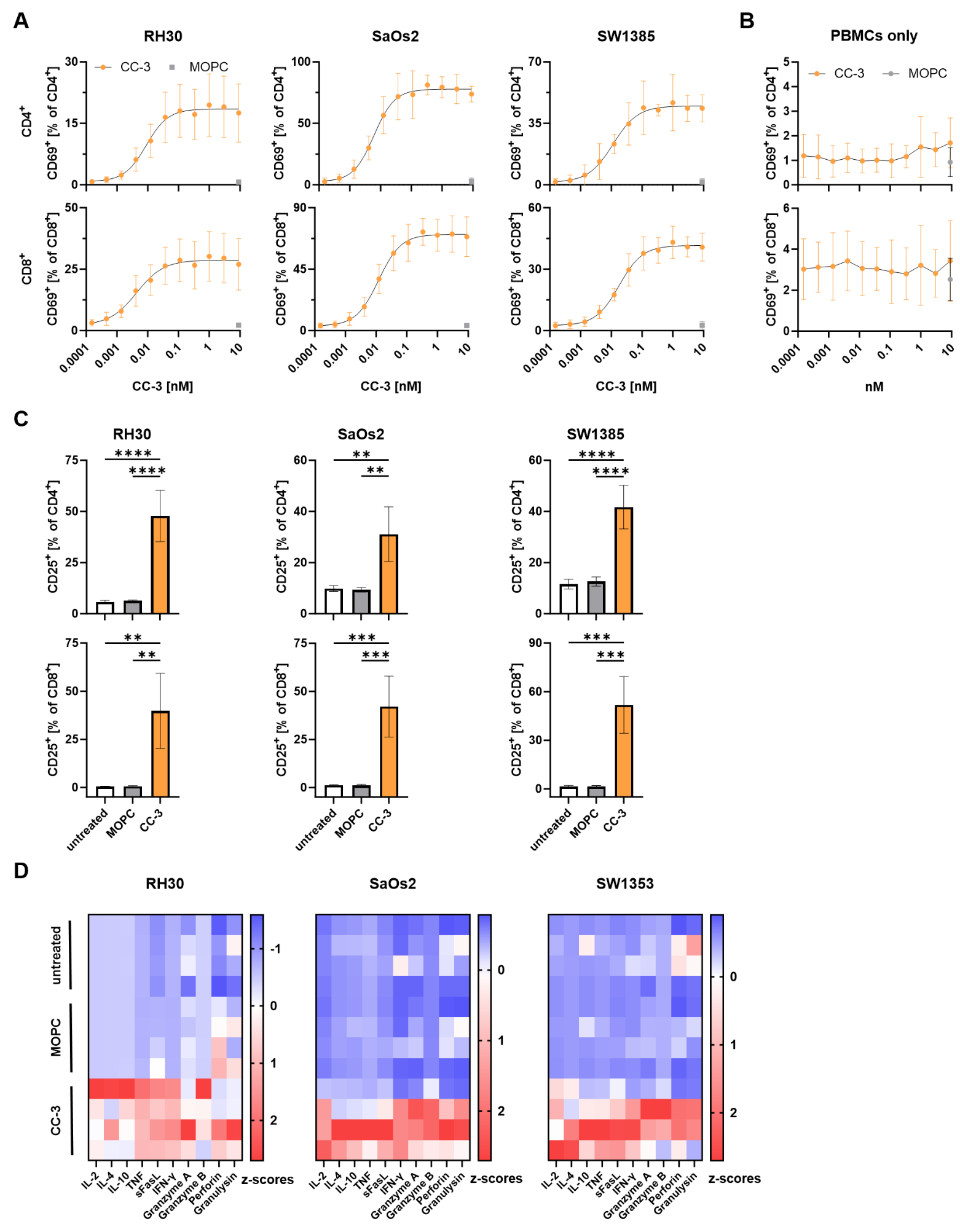


**Supplementary Figure 2** **CC-3-induced T cell activation and cytokine release.** PBMCs (n=4) were incubated with the indicated sarcoma cell lines (E:T 5:1) in the presence or absence of CC-3 or MOPC. Unless otherwise noted, all constructs were used at 1 nM. T cell activation by CD69 and CD25 was assessed for CD4^+^ and CD8^+^ T cells by flow cytometry after 24 h and 72 h, respectively, and secretion of cytokines and effector molecules was determined using Legendplex assays after 24 h. (A) Activation of CD4^+^ (top panels) and CD8^+^ T cells (bottom panels) was determined by CD69 expression. (B) CD4^+^ and CD8^+^ T cell activation without target cells at the indicated concentration of MOPC or CC-3 was assessed by the CD69 expression using flow cytometry. (C) CD4^+^ (top panel) and CD8^+^ (bottom panel) T cells were analyzed for CD25. (D) Cytokine and effector molecules release for the indicated cell lines with or without CC-3 or MOPC control after 24 h was determined using Legendplex assay. The values presented are means ± SD (* p<0.05, ** p<0.01, *** p<0.001, **** p <0.0001).


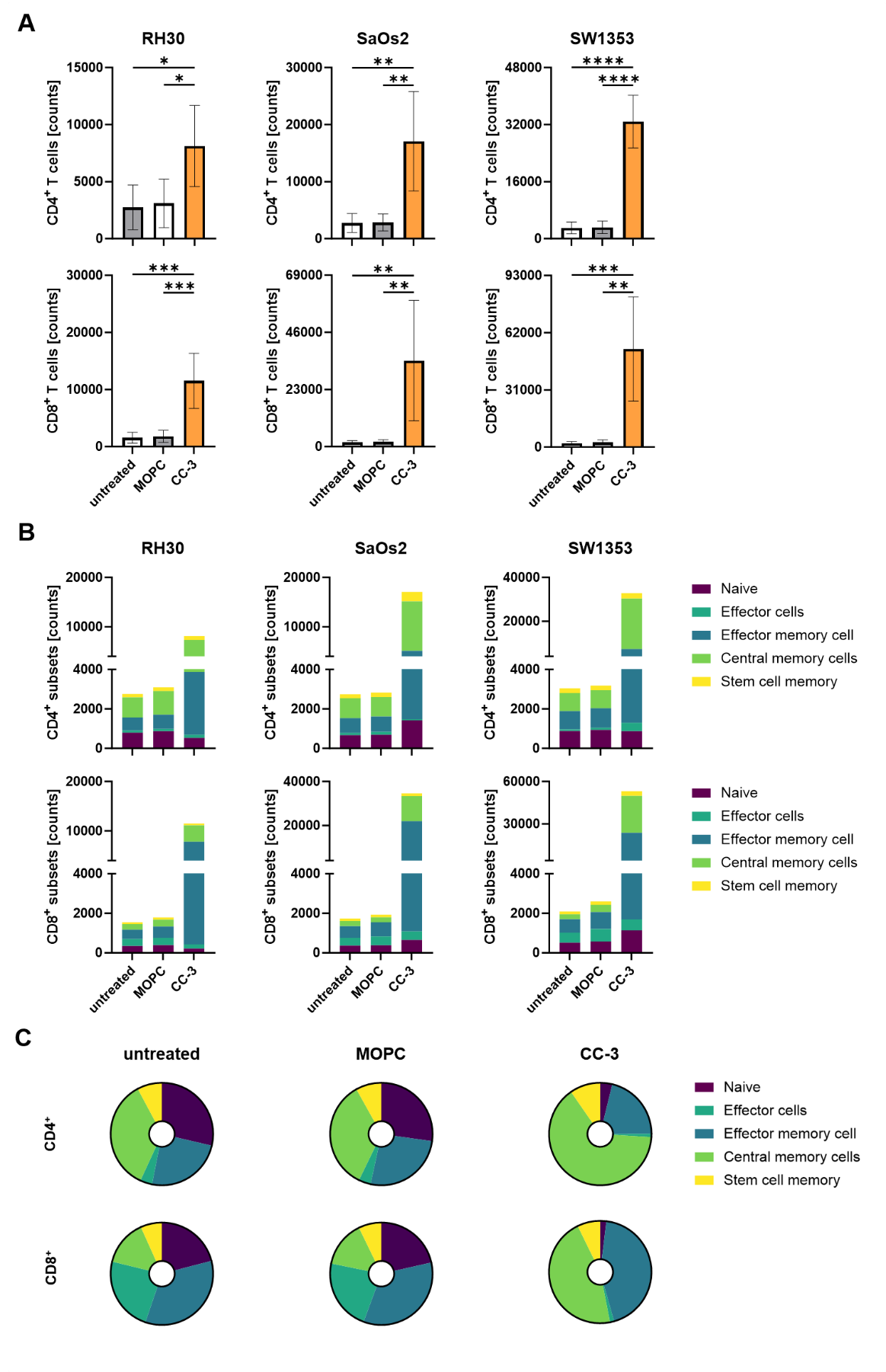
**Supplementary Figure 3 CC-3-induced T cell activation and cytokine release.** PBMCs (n=4) were incubated with the indicated sarcoma cells (E:T 10:1) in the presence or absence of CC-3 or MOPC control (1 nM each) for 6 days. On day 3, PBMC were re-exposed to fresh target cells and the respective treatment for additional 3 days. On day 6, proliferation and T cell subsets were analyzed by flow cytometry for CD62L, CD45RO, CD45RA and CCR7. (**A**) CD4^+^ and CD8^+^ T cell counts for the indicated cell lines are shown. (**B**) Quantification of CD4^+^ and CD8^+^ T cell subpopulations after coculture with the indicated cell lines. (**C**) The distribution of CD4^+^ and CD8^+^ T cells after the indicated treatment for all sarcoma cell lines is shown. The values presented are means ± SD (* p<0.05, ** p<0.01, *** p<0.001, **** p <0.0001).
